# Saudi Journal of Gastroenterology: A Tale of Passion and Perseverance

**DOI:** 10.4103/1319-3767.56086

**Published:** 2009-10

**Authors:** Faisal M. Sanai, Ayman A. Abdo, Ahmad Al Zubaidy, Ibrahim Al Mofleh, Mohammed El Mouzan

**Affiliations:** The Editors, Saudi Journal of Gastroenterology, Riyadh, Saudi Arabia

Someone once said that success is failure turned inside-out. The relevance of those words resonated not too far from our subconscious when, three years ago, the editorial team of the Saudi Journal of Gastroenterology (SJG) sat down to discuss the future of the journal. After 12 years of publishing, SJG was not indexed in any of the major scientific databases and was in a truly dire state of affairs: article submissions were at an all-time low, interest and readership of the journal was flagging and, reflecting a stagnation of purpose, the journal had no electronic version, or an online peer-review system, or a proper scientific publisher.

Contrast this with the present situation. Today we stand at the threshold of success. In July 2009, SJG became indexed in PubMed and also became included in Index Medicus/MEDLINE. In their review of the journal, SJG was considered ‘essential’ reading for clinicians in the field and in the region. This is a very prestigious achievement as only six biomedical journals from Saudi Arabia are listed in this index. This also makes SJG the only indexed gastroenterology/hepatology journal from the Middle-Eastern region. The journal is also archived with PubMed Central, making it compliant with the open access policy of the National Institute of Health, USA.

In 1994, the Board of Directors of the Saudi Gastroenterology Association (SGA), under the pioneering leadership of its first president, Prof. Faleh Al Faleh,[1,2] appointed Professors Mohammed El Mouzan and Ibrahim Al Mofleh as the founding editors of SJG. The task of laying the foundation for a new scientific medical journal was not easy. The challenge was in the struggle, marked by poor resources of a fledgling association and paucity of scientific data from the region. Into this stepped the grit and determination of the editorial team who poured their personal time, effort and resources into the Journal, soliciting articles from leading authors across the world and plying a system unabashedly tilted towards established international journals.

There was no technical help for the journal, no English language editor available, and certainly no wealth of prior experience in journal management. Galley proofs required manual corrections by the editorial team, corrected word-by-word and page-by-page in the most tedious manner imaginable. Grammar, syntax, language, style and even content were all corrected and managed by the editorial team. A determination to succeed was thus the only factor that made the team persevere. Such were the challenges confronted by the editors, from which we can but only take pride in the results they have borne today.

There were some bright sparks, though. First there was the enthusiasm of the Saudi gastroenterology community which included amongst them the philanthropy of Dr. Mohammed Al-Mofarreh who contributed financially and scientifically to the journal, and the benevolence of the Dallah Hospital in Riyadh that provided financial and administrative support during the early years of the journal. But the success of a journal cannot be brought about by individual philanthropy and the interest of a few scientific contributors; a scientific journal must harness the potential to reach out to the greater scientific community at large. And then, only then, can it muster the staying power essential within the community of ever-expanding lists of medical journals.

It was in this scenario that SJG reached out to Medknow Publications, a scientific publishing house which was both, affordable and efficient, and ideally placed to suit the needs of evolving biomedical journals from the developing world. Medknow Publications did not merely serve as publishers for SJG; they fulfilled a parallel role of an advisory body. By joining hands with Medknow, the journal was immediately launched online thereby improving its visibility, and increasing the submission rate from 32 articles in 2006 to 123 in 2007 and poised to reach 400 submissions this year, in effect representing a twelve-fold increase in the submission rate.[3] This is reflected in the increase in journal issues from 3 to 4 per year, while at the same time the article rejection rate has steadily increased from 10% in 2006 to 66% in 2008 clearly suggesting that there has been no compromise in the quality of published articles. This is also evident by the increase in basic science papers published in the journal.

Generally, journals from this region receive submissions from within the region and not from other parts of the world, so the research that we publish is circumscribed by geographical location. While SJG was initially designed to disseminate local and regional literature, this aspect has vastly been superseded by the emergence of the electronic version of the journal. Presently, about 80% of the articles are submitted from outside of Saudi Arabia, with the figure having remained stable over the past three years, thus imparting a truly international flavor to the Journal. For instance, the present issue hosts articles, not just from Saudi Arabia, but also from Iran, Bangladesh, India, Turkey and Kuwait.

Simultaneously, SJG has witnessed other changes that are representative of the evolving reality of the publishing world. The journal has embarked and succeeded in putting all issues from its very inception online. This important step has served investigators who have over the years published important work that was not anymore accessible or available. The journal has also invested heavily to improve visibility in many ways. In testimony of its current visibility, SJG ranks first within a Google search for “Saudi journal” indicative of journal visits online, its popularity within the region, and its overall visibility. It boasts of an international Advisory Board that reads as the who's-who in the world of gastroenterology. The present editorial board has a distinctively ‘young look’ much in line with the journal's policy of encouraging a generation of young investigators. In addition, our list of reviewers has also undergone a paradigm shift with almost 80% of our reviewers now registered from outside of Saudi Arabia.

Amongst other things, the quality of any journal is dependent on the involvement and interest of its reviewers.[4] In this aspect we continue to face many problems. Nothing is more frustrating to the editor than a reviewer who fails to conduct a review, leave alone a proper one, after initially agreeing to the task. Even more frustrating is the fact that authors defy the simple and basic instructions to authors when either submitting or revising their manuscripts, despite the user-friendliness of the manuscript management system. This places an immense burden on the administrative staff, depleting the journal's time, resources and goodwill. Ethical misconduct continues to be a problem[5] although the mechanism in place has made the task of weeding out this problem much simpler. Eventually, a journal is recognized by the quality of its publications. While numbers count, it is quality that eventually matters. With the rising tide of research within the Middle-East, and the developing world in general, we anticipate that we will attract better articles as we move forward.

Finally, our journey at SJG does not end here; it can only begin from here. Our next goal remains to get the journal indexed in the Science Citation Index (SCI) which will provide us with the ‘elusive’ impact factor. The citations received by SJG have increased over the last few years, as can be seen on the journal's website (http://www.saudijgastro.com/showstats.asp?a=tc) and we are hopeful to be included in SCI. Truly, much has been done but, much more needs to be done. The real work begins now and if the passion for success can be a measure of one's achievements, then we, at SJG, are rightly poised to achieve this. As Robert Frost once said: *But I have promises to keep, and miles to go before I sleep* …
